# Effects of Benzodiazepines on Acinar and Myoepithelial Cells

**DOI:** 10.3389/fphar.2016.00173

**Published:** 2016-06-24

**Authors:** Tatiana M. F. Mattioli, Luciana R. A. Alanis, Silvana da Silva Sapelli, Antonio A. S. de Lima, Lucia de Noronha, Edvaldo A. R. Rosa, Yusuf S. Althobaiti, Atiah H. Almalki, Youssef Sari, Sergio A. Ignacio, Aline C. B. R. Johann, Ana M. T. Gregio

**Affiliations:** ^1^Pharmacology and Experimental Pathology, School of Dentistry, Pontifical Catholic University of ParanáCuritiba, Brazil; ^2^Program of Post-Graduation, School of Dentistry, Health and Bioscience School, Pontifical Catholic University of ParanáCuritiba, Brazil; ^3^Department of Oral Pathology, School of Dentistry, Federal University of ParanáCuritiba, Brazil; ^4^Program of Post-Graduation, Department of Pathology, School of Medicine, Pontifical Catholic University of ParanáCuritiba, Brazil; ^5^Department of Pharmacology, College of Pharmacy and Pharmaceutical, The University of ToledoToledo, OH, US; ^6^Program of Post-Graduation, Department of Pharmacology, School of Dentistry, Health and Bioscience School, Pontifical Catholic University of ParanáCuritiba, Brazil

**Keywords:** benzodiazepines, immunohistochemistry, salivary glands, dry mouth, GABA_A_ receptor

## Abstract

**Background:** Benzodiazepines (BZDs), the most commonly prescribed psychotropic drugs with anxiolytic action, may cause hyposalivation. It has been previously shown that BZDs can cause hypertrophy and decrease the acini cell number. In this study, we investigated the effects of BZDs and pilocarpine on rat parotid glands, specifically on acinar, ductal, and myoepithelial cells.

**Methods:** Ninety male Wistar rats were divided into nine groups. Control groups received a saline solution for 30 days (C30) and 60 days (C60), and pilocarpine (PILO) for 60 days. Experimental groups received lorazepam (L30) and midazolam (M30) for 30 days. Another group (LS60 or MS60) received lorazepam or midazolam for 30 days, respectively, and saline for additional 30 days. Finally, other groups (LP60 or MP60) received either lorazepam or midazolam for 30 days, respectively, and pilocarpine for additional 30 days. The expression of calponin in myoepithelial cells and the proliferating cell nuclear antigen (PCNA) in acinar and ductal cells were evaluated.

**Results:** Animals treated with lorazepam showed an increase in the number of positive staining cells for calponin as compared to control animals (*p* < 0.05). Midazolam administered with pilocarpine (MP60) induced an increase in the proliferation of acinar and ductal cells and a decrease in the positive staining cells for calponin as compared to midazolam administered with saline (MS60).

**Conclusion:** We found that myoepithelial cells might be more sensitive to the effects of BZD than acinar and ductal cells in rat parotid glands.

## Introduction

Benzodiazepines (BZDs) are a group of drugs used as anxiolytics, hypnotics, sedatives, muscle relaxants, and anticonvulsants. These drugs have largely been substituted by barbiturates. However, unlike barbiturates, BZDs do not produce a depressive action of the respiratory system and are thus safer and have a greater specificity for treating anxiety symptoms (Howard et al., 2014). BZDs comprise nearly 50% of all prescribed psychotropic drugs (Lader et al., 2009).

BZDs bind at the interface of two subunits (alpha and gamma subunits) of *gamma*-Aminobutyric acid (GABA) receptor type A. It is well-known that GABA is an inhibitory neurotransmitter of the central nervous system (CNS). BZDs facilitate the action of GABA at the GABA_A_ receptors. GABA is a neurotransmitter that has an action in increasing chloride influx, which can lead to hyperpolarization of neurons, thereby inhibiting the generation of an action potential (Howard et al., 2014).

BZDs produce a wide range of adverse effects, including hyposalivation and xerostomia (De Almeida Pdel et al., 2008). Since the last decade, the effect of BZDs in the regulation of salivary secretion, in rat parotid acinar cells, has been widely studied (Okubo and Kawaguchi, 1998). One study has shown that diazepam can decrease salivary secretion by the parotid gland, which involves an inhibitory effect on IP3 and calcium as well as blockade of muscarinic receptor (Kujirai et al., 2002).

It is noteworthy that myoepithelial cells of the salivary glands are located between the basal layer and plasmatic membrane of the secretory acinar cells and interspersed ducts (Klein, 2002). These are characterized by high expression of actin and myosin myofilaments in their cytoplasm, which make their contractility one of their main functions. Myoepithelial cells are highly specialized and possess a double phenotype (smooth epithelial and muscular) and contribute to the drainage from secretory and ductal units. Cells are characterized by the presence of calponin antigen, a protein involved in the system that regulates the contraction of the smooth muscle (Rao et al., 2014).

It is noteworthy that the proliferating cell nuclear anti-antigen (PCNA) is a monoclonal antibody that allows studying the cell kinetics (Wang, 2014) and is therefore a marker of cell proliferation capable of identifying proliferating acinar and ductal cells (Mattioli et al., 2011). Our laboratory has investigated the effect of chronic treatment of BDZ and antidepressant on the parotid glands of Wistar rats and found hyposalivation and hypertrophy of the serous cells. These findings suggest a possible inhibition of the activity of myoepithelial cells stemming from nervous stimulation, a reduction in the number of these cells with chronic use of psychotropic drugs, or a change in the number of acinar and ductal cells (Zaclikevis et al., 2009; Mattioli et al., 2011).

In previous studies (da Silva et al., 2009; Zaclikevis et al., 2009) conducted by our research group, came the question: Since the BZD reduced salivary flow and caused acinar hypertrophy, it was hypothesized that it could be occurring a decrease in mioepitelias cells and/or a reduction of the acinar cells proliferation, respectively evaluated by calponin and PCNA, which is the aim of the present study. In the same studies it also verified that pilocarpine (patent deposited of this research group) reestabished salivary flow which had been reduced with the administration of BZDs. It has been postulated that pilocarpine could then be acting on the myoepithelial cells and/ or interfering with the acinar proliferation, which is also the aim of the present study.

Different drugs selected form because there are the important qualitative and quantitative differences in the pharmacokinetics when comparing midazolam and lorazepam that need to be discussed. Lorazepam is a drug that does not produce active metabolites, and it has a short half-life (12–18 h). By contrast, midazolam contains an active metabolite (hydroxylated derivative) with a half-life of 2 h. The half-life of midazolam is also extremely short (<6 h; De Almeida et al., 2012).

Therefore, the present study aims to investigate the presence of the myoepithelial, and proliferation acinar and ductal cells in rat parotid glands treated with midazolam and lorazepam using immunocytochemistry technique with antibodies against calponin and PCNA. We also investigate the effect of topical administration of pilocarpine in the parotid glands treated with BDZ.

## Materials and method

### Animals

All experimental procedures followed the guidelines of the Didactic-Scientific Vivisection of Animals as well as the Ethical Principles of Animal Experimentation in accordance with Law 6.638 of May 08, 1979 (Goldim, 1985). This study was approved by the Research Ethics Committee at Universidade Tuiuti do Parana (CEP-UTP/55). See more details in Supplementary Materials about Ethical Committee.

This study used 90 male Wistar rats, at a body weight of 250 g, which were provided by the Central Vivarium of the Pontifical Catholic University of Parana (under the supervision of the Animal Committee). Rats were kept under controlled temperature (25° ± 2°C) and relative humidity (50 ± 15%) conditions, normal photoperiod (12 h light/dark cycle). Rats had access to water and *ad libitum* food (Purina™). Animals were housed throughout the experiments in polypropylene cages containing sterile paddy husk (locally supplied) as bedding, with four animals per cage.

### Treatments

The animals were divided into nine groups with ten animals per group. Groups of animals received different treatments (C30, C60, PILO, L30, M30, LS60, MS60, LP60, and MP60) as described in Table 1.

**Table 1:** **Division of the control and experimental groups according to the medication, treatment period, daily dose, and route of administration**.

**Groups**	***n***	**Drugs**	**Treatment period**	**Dose**	**Route of Administration**
1- C30	10	Saline	1–30 days	0.1 mL	Intraperitoneal
2- C60	10	Saline	1–60 days	0.1 mL	Intraperitoneal
3- PILO	10	Pilocarpine^a^	1–60 days	0.05 mL	Topical
4- L30	10	Lorazepam^b^	1–30 days	0.5 mg/kg	Intramuscular
5- M30	10	Midazolam^c^	1–30 days	0.1 mg/kg	Intramuscular
6- LS60	10	Lorazepam	1–30 days	0.5 mg/kg	Intramuscular
		Saline	31–60 days	0.1 mL	Intraperitoneal
7- MS60	10	Midazolam	1–30 days	0.1 mg/kg	Intramuscular
		Saline	31–60 days	0.1 mL	Intraperitoneal
8- LP60	10	Lorazepam	1–30 days	0.5 mg/kg	Intramuscular
		Lorazepam + Pilocarpine	31–60 days	0.05 mL	Intramuscular and topical
9- MP60	10	Midazolam	1–30 days	0.1 mg/kg	Intramuscular
		Midazolam + Pilocarpine	31–60 days	0.05 mL	Intramuscular and topical

*N, sample size*.

a *Orabase gel prepared with 1% pilocarpine hydrochloride (Gerbras Química e Farmacêutica Ltda., São Paulo, Brasil)*.

b *Lorazepam (Cosmética Farmácia de Manipulação Ltda, Curitiba, Paraná, Brazil)*.

c *Midazolam (Cosmética Farmácia de Manipulação Ltda, Curitiba, Paraná, Brazil)*.

### Immunocytochemistry and immunochemical analysis

Parotid glands from male Wistar rats were submerged in paraffin blocks We used tissue microarrays (TMAs) containing ten cylinders of paraffinized salivary glands (3 um in diameters), which were organized in lines and columns (Rocha et al., 2006; Schuler et al., 2008). Each TMA exhibited ten specimens of salivary glands from rats of each group. For immunohistochemistry, anti-calponin, and anti-PCNA (Dako CytomationH; Dako North America, Carpinteria, CA, USA), antibodies were used. The secondary antibody was EnVisionH+Dual Link/Peroxidase (Dako CytomationH), and the peroxidase reactions were revealed with DAB substrate-chromogen system (Dako CytomationH). Slides were counterstained with Harris hematoxylin. We used anti-calponin monoclonal antibody at 1:800 for myoepithelial cell staining and anti-PCNA antibody at 1:400 for staining of proliferating acinar and ductal cells. PCNA is a monoclonal antibody that allows the study of cell kinetics.

Sections were visualized by only one examiner using an OlympusH BX50 optical microscope (Olympus Corporation, Ishikawa, Japan) with a 40X objective. Expression of calponin in the myoepithelial cells and PCNA in the acinar and ductal cells were analyzed using the entire TMA area. The presence or absence of staining of PCNA and calponin antibodies was evaluated. Cells that exhibited expression for PCNA in the glandular epithelium were considered positive (represented by a brownish staining), regardless of the staining intensity. To analyse the positivity to calponin, the identification of the myoepithelial cells was based on their morphology and location followed described associated with a cytoplasmatic brownish staining. Also the observer was calibrate, due to that only marking the cells in which the nucleus was considered. Myoepithelial cells are normal constituent of the salivary acini and smaller ducts, and are found between the epithelial cells and the basement membrane. Microscopic examination shows that myoepithelial cells are thin and spindle-shaped and situated between the basement membrane and epithelial cells (Balachander et al., 2015).

There were 24 histological fields in each cylinder of paraffinized tissue. Before counting the number of stained cells, we verified the number of fields (out of 24). Therefore, the integrity and quality of the tissue as well as the presence of technical artifacts were taken into consideration. After evaluating the 24 histological fields of all cylinders, the numerical indices for the positive staining of cells for calponin and for PCNA were obtained.

To determine the average value of stained cells for calponin and PCNA from each cylinder, the average value of stained cells in a cylinder was represented by the sum of the number of stained cells for the antibody in each evaluated field divided by the number of evaluated fields. After obtaining the average value for each cylinder, the values were added together and divided by 10 (equivalent to the total number of TMAs), which resulted in the average value of stained cells for each group. See more details in Supplementary Materials about histological analysis.

In addition to calponin and PCNA variables, this study also used the average values for the stimulated salivary flow rate (SSFR) and cellular volume (CV) as determined in a study by Rinaldi et al. (2015). Because the present study investigated the same sample as was performed previously (Rinaldi et al., 2015), the SSFR and CV findings were compared to the immunohistochemical staining results for calponin and PCNA.

### Statistical analysis

Data were analyzed using SPSS software. Normality analysis was performed using the Kolmogorov-Smirnov test. The Levene test was used to analyze the variance of homogeneity. For the groups with a normal distribution, analysis of variance (ANOVA) at one criterion was performed. When ANOVA at one criterion showed differences among the groups and treatment, the Tukey HSD multiple comparison test was used for variables that presented variance of homogeneity among the groups. For the variables that did not present variance homogeneity, the Games-Howell test was used. The level of significance for all statistical tests was set at 5% (*p* < 0.05). The analyzed variables were calponin and PNCA, which were compared to the CV and SSFR variables (Rinaldi et al., 2015). See more details in Supplementary Materials about statistical analysis.

## Results

**Figure 1:**
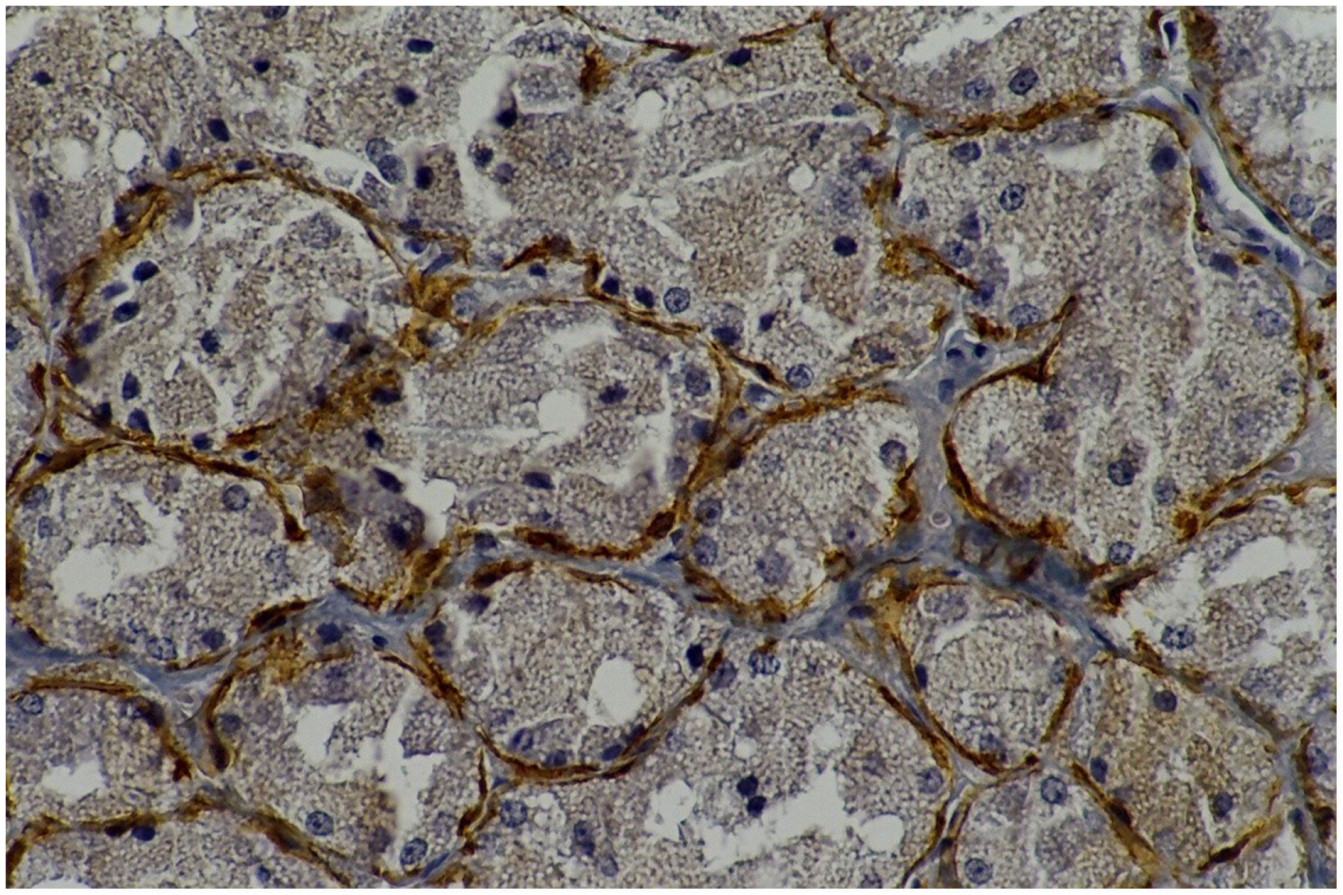
**Rat parotid gland submitted to 30 days with lorazepam (L30) showing myoepithelial cells in brown (immunohistochemical staining of calponin, original magnification 400 x)**. L30 showed higher number of myoepithelial cells when compared to C30.

**Figure 2:**
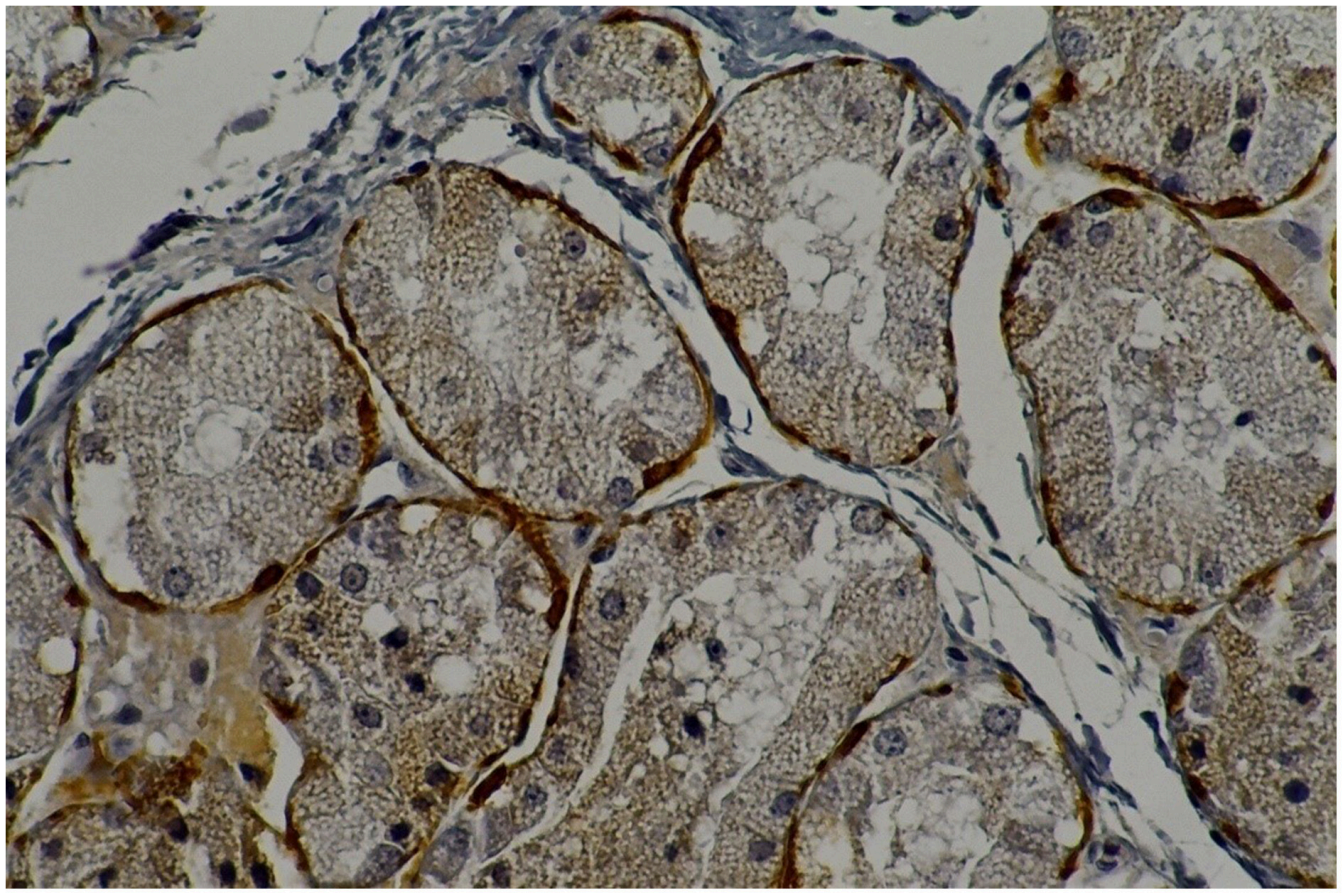
**Rat parotid gland submitted to 30 days with saline (C30) showing myoepithelial cells in brown (immunohistochemical staining of calponin, original magnification 400 x)**.

L30 and M30 showed lower values to SSFR (Graphic 1 and Table 2 respectively) and higher to CV (Graphic 2 and Table 2) when compared with C30. L30 showed higher value to number of C when compared to C30 (Graphic 3, Figures 1, 2). None differences to PCNA was observed comparison L30 and M30 to C30 (Graphic 4 and Table 2).

**Graphic 1:**
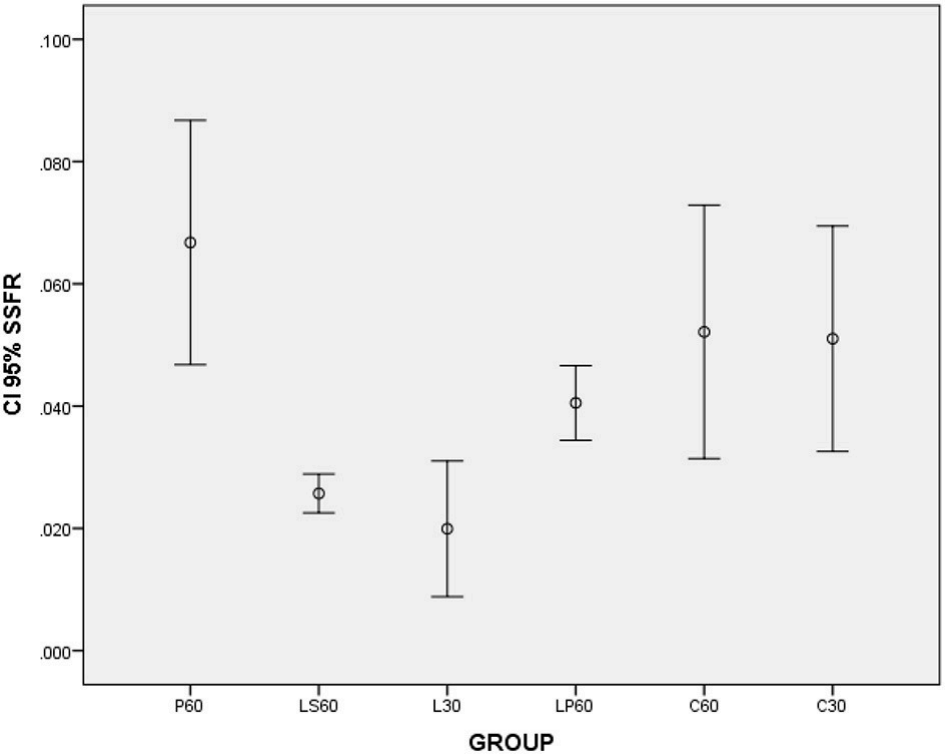
**Box-plots to Stimulated Salivary Flow Rate (SSFR - mL/min) according to the groups treated for: 30 and 60 days with saline (C30 and C60 respectively); pilocarpine (P60), lorazepam (L30), lorazepam and saline (LS60), and lorazepam and pilocarpine (LP60)**. Comparisons with *p* < 0.05: L30 × C30; P60 × LS60; LS60 × LP60. CI 95% = 95% confidence interval. Data obtained from findings reported by Rinaldi et al. (2015).

**Table 2:** **Average values, standard deviation, and *p*-value studied according to the groups treated for 30 days with saline (C30), and midazolam (M30)**.

**Groups**	**C30**		**M30**	
	***X***	***DP***	***X***	***DP***
SSFR (mL/min)^δ^	0.051 ± 0.025^a^		0.019 ± 0.009^a^	
CV (μm3)^δ^	6965.68 ±3792.96^a^		20036.56 ± 7955.70^a^	
C	13.9 ± 2.6		15.3 ± 7.1	
PCNA	55.8 ± 14.4		39.6 ± 30.0	
				

δ *Values obtained from findings reported by Rinaldi et al. (2015); SSFR (mL/min), Stimulated salivary flow rate in millimeters per minute; CV, Cell volume in square micrometers; PCNA, Proliferating Cell Nuclear Antigen; C, Antibody against calponin antigen; X, average; SD, standard deviation;p, p-value of ANOVA;Similar letter represents statistically significant difference between the groups*.

**Graphic 2:**
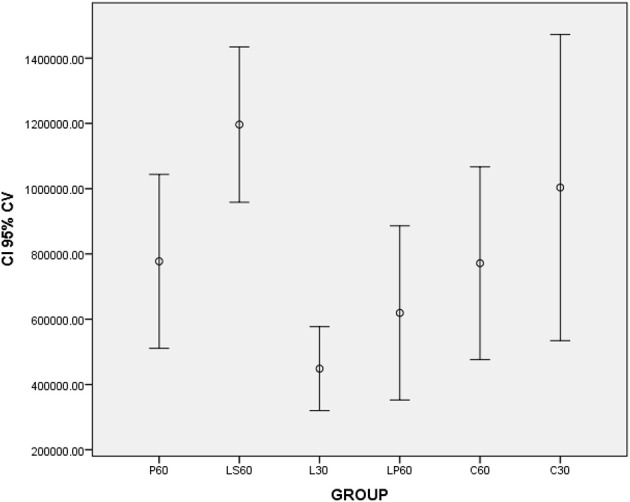
**Box-plots to Cell Volume (CV -μm^3^) according to the groups treated for: 30 and 60 days with saline (C30 and C60 respectively); pilocarpine (P60), lorazepam (L30), lorazepam and saline (LS60), and lorazepam and pilocarpine (LP60)**. Comparisons with *p* < 0.05: L30 × C30; P60 × LS60; C60 × LS60. CI 95% = 95% confidence interval. Data obtained from findings reported by Rinaldi et al. (2015).

**Graphic 3:**
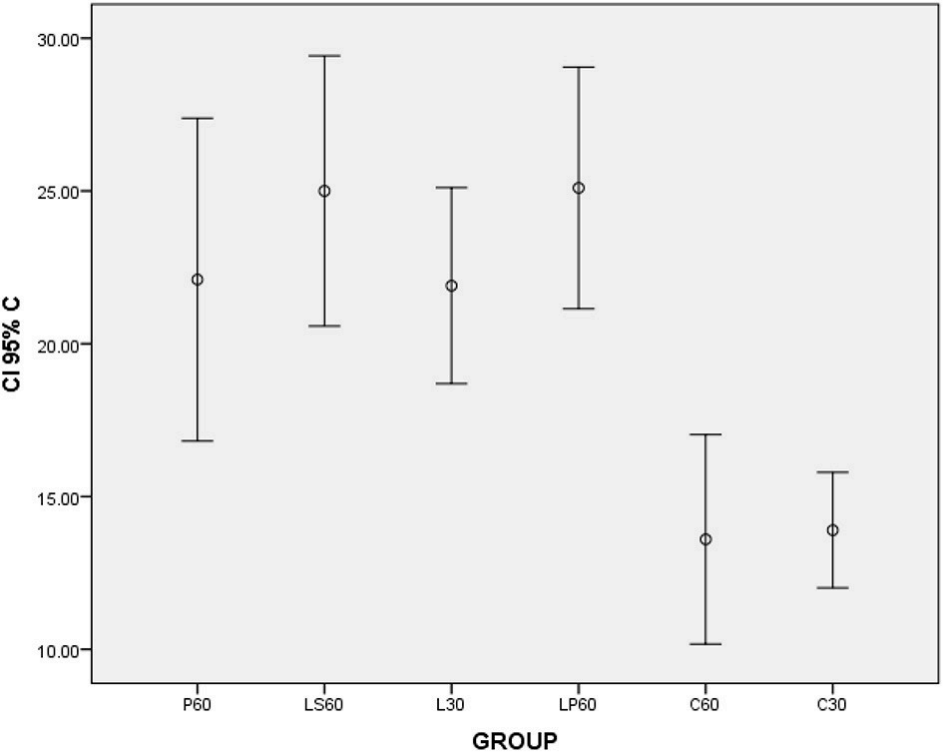
**Box-plots to calponin index (C) according to the groups treated for: 30 and 60 days with saline (C30 and C60 respectively); pilocarpine (P60), lorazepam (L30), lorazepam and saline (LS60), and lorazepam and pilocarpine (LP60)**. Comparisons with *p* < 0.05: L30 × C30; LP60 × C60. CI 95% = 95% confidence interval.

**Graphic 4:**
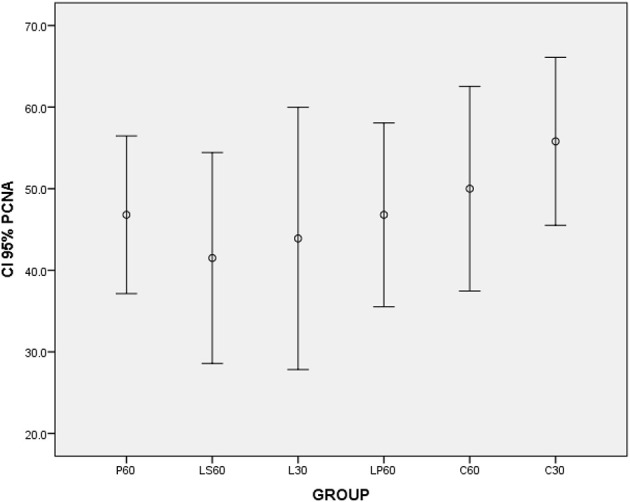
**Box-plots to Proliferating Cell Nuclear Antigen index (PCNA) according to the groups treated for: 30 and 60 days with saline (C30 and C60 respectively); pilocarpine (P60), lorazepam (L30), lorazepam and saline (LS60), and lorazepam and pilocarpine (LP60)**. None differences were observed. CI 95% = 95% confidence interval.

**Figure 3:**
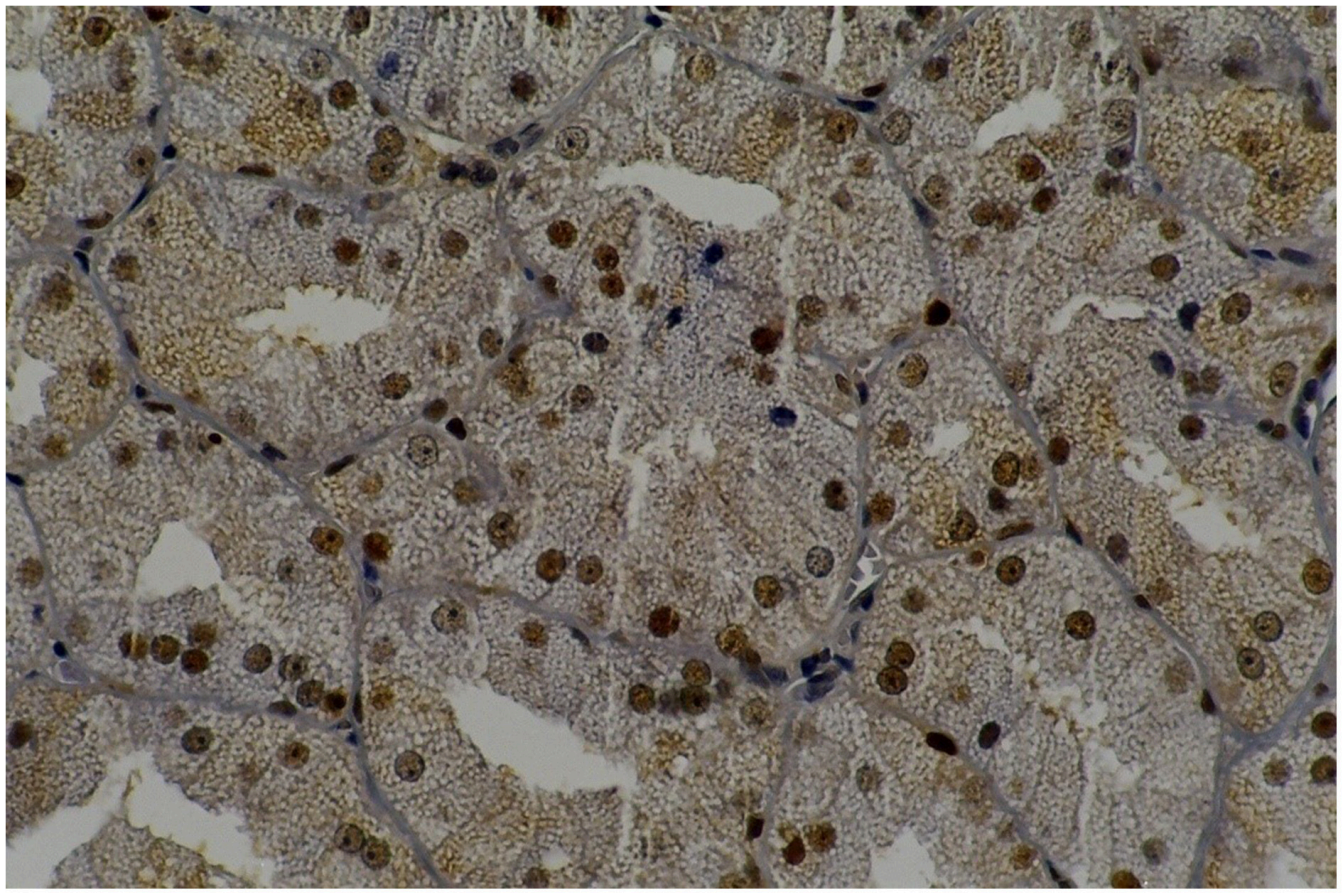
**Rat parotid gland submitted to pilocarpine with midazolam (MP60) showing acinar and ductal cells in proliferation in brown nuclei (immunohistochemical staining of PCNA, original magnification 400 x)**. MP60 showed higher PCNA as compared to MS60.

**Figure 4:**
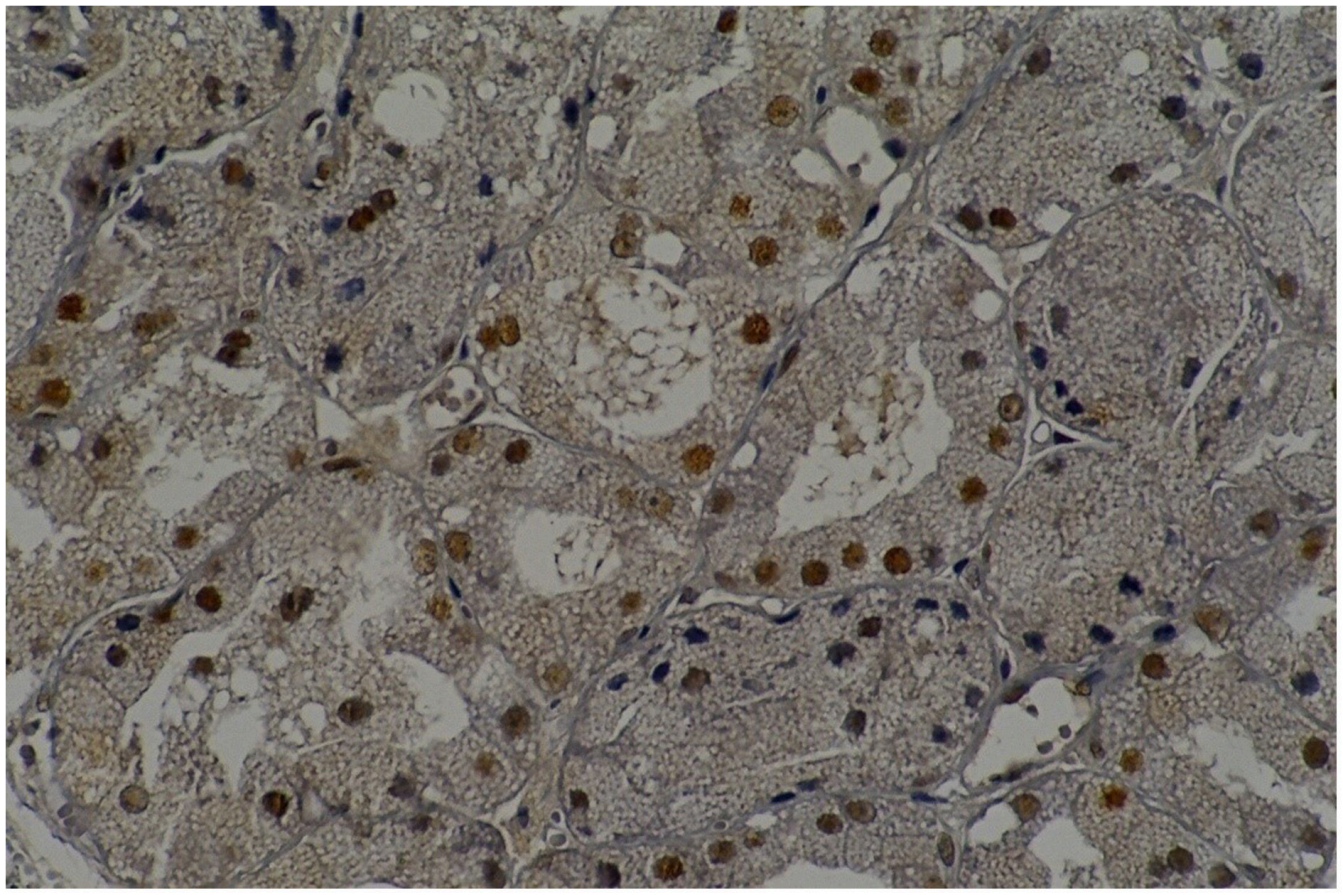
**Rat parotid gland submitted to midazolam and saline (MS60) showing acinar and ductal cells in proliferation in brown nuclei (immunohistochemical staining of PCNA, original magnification 400 x)**.

LP60 and MP60 showed similar values to SSFR (Graphic 1 and Table 3) when compared to C60. None differences to SSRF was observed comparison LS60 or MS60 to C60 (Graphic 1 and Table 3). LS60 or LP60 revealed higher values to C as compared to C60 (Graphic 3). MP60 showed higher PCNA (Figures 3, 4) and lower C as compared to MS60 (Table 3).

**Table 3:** **Average values and standard deviation of studied variables according to the groups treated with pilocarpine (P60), saline (C60), midazolam and saline (MS60), and midazolam and pilocarpine (MP60)**.

**Group**	**P60**		**C60**		**MS60**		**MP60**	
	***X***	***DP***	***X***	***DP***	***X***	***DP***	***X***	***DP***
SSFR (mL/min)^δ^	0.066 ± 0.027^a^		0.052 ± 0.028		0.022 ± 0.005^a^		0.038 ± 0.028	
CV (μm3)^δ^	5.825.42 ± 1.968.07^ab^		6.505.56 ± 3.343.48^c^		8.143.93 ± 3.470.78^a^		9.152.06 ± 4.605.78^bc^	
C	21.1 ± 7.385^a^		13.6 ± 4.788^a^		15.6 ± 6.221^b^		14.3 ± 6.147^b^	
PCNA	46.8 ± 13.505		50 ± 17.530		39 ± 14.110^a^		67. ± 19.102^a^	
								

δ *Values obtained from findings reported by Rinaldi et al. (2015); SSFR (mL/min), salivary flow stimulated in millimeters per minute; CV, Cell volume in square micrometers; PCNA, Proliferating Cell Nuclear Antigen; C, Antibody against calponin antigen; X, average; SD, standard deviation; p, p-value of ANOVA; Similar letter represents statistically significant difference between the groups*.

## Discussion

Chronic use of lorazepam and midazolam for 30 days induced a reduction in the SSFR and an increase in the CV in rat parotid glands. Lorazepam, when administered for 30 days (L30 group), increased the number of myoepithelial cells when compared to the saline group (C30). When pilocarpine was administered with lorazepam or midazolam for 60 days, pilocarpine attenuated the deficit in SSFR. Administration of lorazepam with saline solution (LS60) or pilocarpine (LP60) caused an increase in the number of myoepithelial cells as compared to the groups that received only saline solution (C60). When pilocarpine was administered with midazolam (MP60), there were an increase in the proliferation of acinar and ductal cells and a reduction in the number of myoepithelial cells as compared to the groups that received midazolam and saline (MS60).

Chronic treatment with lorazepam and midazolam for 30 days induced reduction of SSFR in rats. In groups treated for 30 days, the reduction of SSFR was more pronounced than in groups treated for 60 days. It is important to note that in the groups treated for 60 days (MS60 and LS60), administration of medication were suspended at the final 30 days and after this period the animals received only saline. It is possible that this may allow for a reorganization of the physiology of salivary secretion in the parotid glands. To investigate these findings further, some experiments are warranted to be tested, including pharmacokinetics and pharmacodynamics of BZDs, determination of the presence of peripheral receptors in the salivary glands (Sawaki et al., 1995), physiological adaptation when using psychotropic drugs, change in the sensitivity of the receptors during chronic use of psychotropics, and the activity of the myoepithelial, acinar, and ductal cells.

BZDs act selectively in GABA_A_ receptors, which intensify GABA response, making it easier to open the chloride channel (Zaclikevis et al., 2009). The activation of GABA_A_ receptors increases conductance to the chloride ion, which is found in greater concentrations on the external surface of the membrane. As the chloride flow in the concentration gradient, hyperpolarization of the post-synaptic membrane occurs, (Zaclikevis et al., 2009). In this manner, an intensification of the inhibitory activity of the CNS occurs when BZD is administered.

GABA_A_ receptors can be both central and peripheral (located in the salivary glands) (Okubo and Kawaguchi, 2013). Kosuge et al. (2009) demonstrated the presence of peripheral GABA_A_ receptors in the acinar cells of parotid and submandibular glands of rats. The expression of receptors in the salivary glands is less than what was found in the CNS (Howard et al., 2014).

Numerous studies, both on human and animals, have demonstrated that BZDs cause a reduction in salivary flow, with severe oral side effects (De Almeida Pdel et al., 2008; Femiano et al., 2008). BZDs act to induce reduction in salivary flow through GABA_A_ receptors located in the salivary glands as well as through indirect actions of the CNS on the salivary glands mediating central GABA_A_ receptors (Kujirai et al., 2002; Okubo and Kawaguchi, 2013). In addition to inhibiting the muscarinic receptors present in the salivary glands, which are responsible for the salivary flow, BZDs also can affect the transport of chloride and the calcium influx, which can lead to fluid secretion. As a result, a reduction in the salivary secretion activity occurs. BZDs can also inhibit β-adrenergic receptors, in turn blocking the release of amylase from rat parotid glands (Okubo and Kawaguchi, 2013).

The present study demonstrated that when the treatment with BZDs extended for 60 days, the reduction in SSFR was found to be not significantly different when compared to the control group. It is known that prolonged use of psychotropic drugs is responsible for changes in the sensitivity of the receptors (Howard et al., 2014). The therapeutic and collateral effects of a drug gradually diminishes when administered in a continuous or repeated manner, which can in turn cause desensitivity, resistance, or tolerance to the drug. A physiological adaptation can also occur. Hence, it is common to observe that both therapeutic and collateral effects of drugs tend to diminish with the duration of administration, despite the continuous use of the drug (Howard et al., 2014). Thus, it can be concluded that the weakest reduction of SSFR in rats treated for 60 days occurred due to the effects of tolerance and adaptability of the psychotropic drugs.

The absence of a statistically significant difference in the SSFR among the C60, MS60, and LS60 groups suggests that there was in fact a reestablishment of normal SSFR 30 days after administration of the drugs (Table 3). However, these data must be analyzed carefully, given that the reduction of SSFR of more than 50% lead to it relevant detrimental clinical consequences (C60—0.052 mL/min; MS60—0.022 mL/min).

One of the main effects of anxiolytics and hypnotics is the myorelaxant action. The myoepithelial cells, though classified as non-muscular cells, present an accentuated capacity of contraction. The cytoplasm contains numerous contractible actin and myosin microfilaments, intermediary filaments from the cytokeratin 14 family, and typical structures of smooth muscle fibers (Rao et al., 2014). The myoepithelial cells perform contraction functions, contributing to the drainage of the secretion from the secretory and ductal units (Rao et al., 2014). Thus, it is suggested that the myorelaxant activity caused by the anxiolytics can be, in part, responsible for the reduction of the SSFR of rats treated with lorazepam and midazolam.

The chronic use of lorazepam for 30 days (L30) and 60 days (LS60 and LP60) induced a significant increase in the number of myoepithelial cells when compared to the saline groups (C30 and C60). For the groups that received midazolam (M30, MS60, and MP60), this difference did not occur (Tables 2, 3). Here, the important qualitative and quantitative differences in the pharmacokinetics when comparing midazolam and lorazepam need to be discussed. Lorazepam (examples of commercial names: Lorax®, Mesmerin®) is a drug that does not produce active metabolites. It is metabolized through its direct connection with a glycoside radical, a process that is faster than oxidation. In addition, lorazepam has a short half-life (12–18 h).By contrast, midazolam (examples of commercial names: Dormonid®, Dormire®) contains an active metabolite (hydroxylated derivative) with a half-life of 2 h (De Almeida et al., 2012). The half-life of midazolam is also short (<6 h). Considering that lorazepam has a half-life of up to three times as long as midazolam, its effects last longer, which can explain the significant changes in the salivary glands. BZDs, as liposoluble drugs, are released through the salivary flow and can circulate for a prolonged time in the saliva (De Almeida et al., 2012).

The myoepithelial cells have a contractile function, are involved in aiding the salivary secretion, and are located around the acinar cells and in the interspersed portion of the ductal cells (Rao et al., 2014). It is valid to speculate that in an activity to compensate for the lack of saliva, the organism, in an attempt to increase the activity of the myoepithelial cells to expel the retained saliva, induces an increase in the number of these cells so that they, in their function of contractility, aid in the release of the retained saliva. Burgess et al. (1996) showed, through immunohistochemical studies performed on atrophic parotid glands of rats caused by ductal cell obstruction, that the myoepithelial cells are capable of proliferating, especially when caused by an injury to the gland itself. This finding suggests that lorazepam may well be able to act as a chemical agent capable of unleashing mitoses and, consequently, increasing the number of myoepithelial cells.

In the present study, the administration of pilocarpine associated with lorazepam over a 60-day period was able to reestablish the normal SSFR (C60, LS60, and LP60 groups), in turn proving its cholinergic agonist capacity. As it presents a cholinomimetic action, the pilocarpine activates the M3 muscarinic receptors present in the salivary glands, promoting an increase in the release of salivary secretion. It is still rather unclear exactly how these mechanisms work. Kujirai et al. (2002) reported that diazepam was capable of producing a reduction in the SSFR, and in the acinar cells of the rat parotid glands, diazepam induced an acceleration of the chloride influx as well as an inhibition of the efflux.

When pilocarpine was associated with midazolam, an increase in the proliferation of acinar and ductal cells and a reduction of the number of myoepithelial cells were observed when compared to the group that received midazolam and saline solution. This may be the consequence of a pharmacological synergism or of pharmacodynamics between midazolam and pilocarpine. It is well-known that the association of the two drugs can result in a pharmacological synergism, in which the combined effect of the two substances proves to be greater than the sum of each one separately (Zaclikevis et al., 2009; De Almeida et al., 2012). On the other hand, pharmacodynamics results from the action of the drugs involved in the same receptor. One drug can increase the effect of the agonist by stimulating the receptivity of its cellular receptor or by inhibiting the enzymes that inactivate it in the location where the action takes places. The reduction in effect may be due to the competition for the same receptor, with the agonist having a greater affinity (Zaclikevis et al., 2009; De Almeida et al., 2012).

Ouchi et al. (2011) evaluated the modulation of GABA_A_ receptor, adrenoceptor, and muscarinic receptor by diazepam in rat parotid glands. This study suggested that uninterrupted administration of diazepam changes the connection for β-adrenoceptor and GABA_A_ receptor binding sites in parotid gland membranes, and changes in these binding sites may be correlated to the diazepam-induced inhibition of salivary secretion. This study demonstrated that the group of animals that received BZDs for 1 month showed a reduction in the number of proliferating acinar cells, as verified by PCNA staining. These results have contributed to the hypothesis that BZDs do in fact promote suppression in the parotid gland.

Rinaldi et al. (2015) confirmed that chronic administration of midazolam and lorazepam reduced the number of acinar cells in the parotid glands of rats, which may have contributed to the reduction of salivary flow. The association of midazolam with pilocarpine led to the reestablishment of acinar cells, which may have favored the restoration of the salivary flow, as shown above. Once again, the suppression action of BZDs on the salivary glands was observed, in turn reducing the salivary flow; however, pilocarpine has contributed to a reversal effect.

Okubo and Kawaguchi (2013) carried out an important investigation on anxiolytics, BZDs, and salivary glands. This study used a rat submandibular gland perfusion method to investigate the suppressor regulation of the GABA_A_ receptor and showed that salivary secretion is inhibited by bicuculline, a GABA(A)-receptor [GABA(A)-R] antagonist, in rat salivary glands. This finding represents one step toward a better understanding of the role of GABA_A_ receptor in the salivary gland, reinforcing the results from the present study, which confirmed that when anxiolytics (lorazepam and/or midazolam) were administered together with pilocarpine (a cholinergic agonist), a reversal of the suppression of the release of the salivary secretion could be observed, including a restorative action of the salivary flow.

A potential limitation of our analysis reside in the fact that, although the utmost care was taken to ensure a proper calibration, the analysis of the histological data were perfomed by a single observer.

Our eye-opening study investigated how pilocarpine, a cholinergic agonist, may act in the GABA_A_ receptor on the salivary gland.

## Conclusion

The histomorphometric revealed that, after the long-term treatment anxiolytics drugs: increase the cellular volume (hypertrophy acinar), we suggested that it could be due to blockage of the stimulus on the cholinergic receptors of the parotid glands contributed to the reduction in salivary flow.

The effects of Midazolam per 30 days: reducing the salivary flow rate, increase Cell volume, results of immunohistochemistry the work revealed that: Calponin showed no interference on myopithelial cell neither PCNA.

The main results is that myoepithelial cells proved to be more sensitive to the effects of BZDs than acinar and ductal cells in rat parotid glands.

The effects of Midazolam per 60 days: Midazolam plus Pilocarpine showed a tendency to increase the salivary flow rate when compared with only Midazolam, Midazolam plus Pilocarpine not increase the number of myopithelial cell, neither Midazolam plus saline, but Midazolam plus Pilocarpine increase of proliferating acinar and ductal cell when compared with only Midazolam.

The effect of Lorazepam per 30 days: reducing the salivary flow rate, increase Cell volume, results of immunohistochemistry the work revealed that: Calponin showed increase the number of myopithelial cell, but none interference of PCNA.

The effect of Lorazepam per 60 days: Lorazepam plus Pilocarpine increase the salivary flow rate when compared with only Pilocarpine, Lorazepam plus Pilocarpine increase the number of myopithelial cell, but none interference of PCNA.

On the other hand, the treatment with Pilocarpine led to the reestablishment of the number of myoepitelial cells on the Midazolam plus Pilocarpine, which may have favored the restoration of salivary flow rate. This is the main action of Pilocarpine.

The chronic administration per 30 days of Lorazepam and Midazolam proved to **be able** of decrease salivary flow rate, but there are some difference between these groups, once Lorazepam has long bioavailability when compare Midazolam (short bioavailability) and pharmacokinetic and pharmacodynamic differences justifying the different results shown by these BZDs.

## Author contributions

Participated in research design: LA, AG, Conducted experiments: LA, TM, SS, AG Contributed new reagents or analytic tools: LN, Performed data analysis: SI Wrote or contributed to the writing of the manuscript: LA, TM, SD; AJ, AL, ER, YA, AA, YS, AG. AG: This is the mentor of research. She is a Pharmacologist and interpretation of data to respect drugs. She has participated in research design, conducted experiments, wrote and contributed to the writing of the manuscript.

### Conflict of interest statement

The authors declare that the research was conducted in the absence of any commercial or financial relationships that could be construed as a potential conflict of interest.

## Abbreviations

BZDs: Benzodiazepines
C 30: Saline for 30 days
C60, Saline for 60 days: PILO pilocarpine
L30: Lorazepam for 30 days
M30: Midazolam 30 days
LS60: Lorazepam 30 days + Saline 30 days
MS60: Midazolam 30 days + Saline 30 days
PCNA: proliferating cell nuclear antigen
SSFR: stimulated salivary flow rate
CV: cellular volume
GABA: Gamma-aminobutyric acid
TMAs: tissue microarrays.
