# Supplementary material for: Effects of Benzodiazepines on Acinar and Myoepithelial Cells
Source: Front Pharmacol. 2016 Jun 24;7:173. doi: 10.3389/fphar.2016.00173 (PMC4919344; doi:10.3389/fphar.2016.00173)
Supplement: Supplementary file 1 [file Table1.PDF]

REPRODUCIBILITY POWER OF MEASURED VALUES

Initially within each group constituted of n = 10 measurements, the values are sorted and made a random selection of n = 5, therefore comprised a sample of n = 30 that was measured again by the same examiner in a 30 day interval blindly.

Table 1 is the total variance, the variance of the error, the error variance in the total variance (Dalberg Error), and the coefficient of reliability and the Pearson correlation coefficient for PCNA and C variables measures carried out in two stages. In the analysis of Dahlberg error, followed by the study HOUSTON (1983) and MIDTGARD et all (1974), suggesting that mode, ideal, the variance of the error should not exceed 5% of the total variance. In cases where the error variance is greater than 8%, the measurement would be inappropriate. It was observed that the Dahlberg error did not exceed 2.8% for the two variables, indicating that the evaluator reproduced the measures acceptably.

TABLE 1 - TOTAL VARIANCE, ERROR VARIANCE, DAHLBERG ERROR, RELIABILITY COEFFICIENT AND PEARSON CORRELATION COEFFICIENT FOR VARIABLE PCNA and C

VARIABLE	VARIANCE TOTAL St^2	VARIANCE ERRO Se^2	DAHLBERG ERROR (%)	CONFIAB. COEF. (%)	CORREL COEF.. (%)
PCNA	257.0885	3.6667	1.4262	98.5738	98.6640
C	52.0305	1.4500	2.7868	97.2132	97.2452

SOURCE: RESEARCH SOURCE

After calculating the Dahlberg reliability coefficient applied the Student t test for paired samples, to evaluate if the researcher has committed systematic error, that is, at this moment 2 measurements were underestimated or overestimated. As can be seen in Table 2, there was no statistically significant difference in mean values of two variables between times 1 and 2, since $p > 0.05$, indicating no systematic error.

TABLE 2 - STUDENT T TEST FOR SAMPLES PARED

Variable	Average	n	Standard deviation	Statistic	P Value
PCNA1	44.166667	30	15.9159069	1.665205203	0.1066405
PCNA2	43.366667	30	16.1511894		
C1	19.3000	30	7.04738	0.529588724	0.6004283
C2	19.1333	30	7.37532		

Source: Research Source

We would like to have performed the measurements between examiners, as suggested by the reviewer (a practice to be adopted for future work), but as it's been a long time since the immunohistochemical reaction and over time the intensity of color of the marking undergoes change, counting by the second appraiser at the present time it could not be compared to the first observer made at an earlier time. However the above results show that the appraiser was properly calibrated, thereby reproduce reliably measures, ensuring that the assessment is not biased by the judgment of an individual operator.

Besides that, this work is part of a big project that was done long time ago. Then, we are thinking about to do the new count is not possible, because of this long time could produced damage in the intensity of the marking.

HOUSTON, W. J. B. The analysis of error in orthodontic measurements. **Am J Orthod**, St Louis, v.83, n.2, p.382-390, May, 1983.

MIDTGARD, J.; BJÖRK, G.; LINDER-ARONSON, S. Reproducibility of cephalometric landmarks and errors of measurements of cephalometric cranial distances. **Angle Orthod**, Appleton, v.4, n.1, p.56-62, Jan. 1974.
