# Supplementary material for: Effects of Benzodiazepines on Acinar and Myoepithelial Cells
Source: Front Pharmacol. 2016 Jun 24;7:173. doi: 10.3389/fphar.2016.00173 (PMC4919344; doi:10.3389/fphar.2016.00173)
Supplement: Supplementary file 2 [file DataSheet1.PDF]

Animal Housing

All the experimental procedures followed the guidelines of the Didactic-Scientific Vivisection of Animals as well as the Ethical Principles of Animal Experimentation in accordance with Law 6.638 of May 08, 1979 (GOLDIM, 1985). This study was approved by the Research Ethics Committee at Universidade Tuiuti do Parana (CEP-UTP/55).

This study was made with 90 male Wistar rats (Rattus norvegicus albinus, Rodentia, mammalian) ca 250g, were provided by the Central Vivarium of the Pontifical Catholic University of Parana (under the supervision of the Animal Committee). The rats were kept under controlled temperature (25°±2°C) and relative humidity (50%±15%) conditions, normal photoperiod (12hrdark:12hrlight) , with drink able water and food (Purina™) available *ad libitum* . The animals were housed throughout the experiment in polypropylene cages containing sterile paddyhusk (locally supplied) as bedding, with four animals per cage.

Thus, ninety animals were distributed among nine groups of ten animals. Groups of animals received different treatments (C30, C60, PILO, L30, M30, LS60, MS60, LP60, and MP60) are described in Table 1.

ANEXO 1 – APROVAÇÃO DO COMITÊ DE ÉTICA

Universidade
Tuiuti
do Paraná

PROPPE
PRÓ-REITORIA DE PÓS-GRADUAÇÃO, PESQUISA E EXTENSÃO

Curitiba, 26 de agosto de 2003

Of. CEP-UTP n.º 55 /2003

Sra. Pesquisadora

O Comitê de Ética em Pesquisa da Universidade Tuiuti do Paraná, CEP-UTP, após apreciação do Projeto de Pesquisa, de sua autoria, intitulado **"Efeito do pilocarpus jaborandi sobre a xerostomia induzida por drogas psicotrópicas"** considerou-o **APROVADO.**

Prof.ª Dr.ª BEATRIZ HELENA SOTTILE FRANÇA
Coordenadora do CEP-UTP

Ilmo. Sra.
Ana Maria Trindade Grégio
Pesquisadora Responsável

ANEXO 2 – PROJETO APROVADO PELO CNPq

CNPq

Diretoria de Programas Programas Temáticos e Setoriais
Coordenacao-Geral do Programa de Pesquisa Em Saude
Coordenacao do Programa de Pesquisa Em Saude
PROGRAMA BASICO DE ODONTOLOGIA

Luciana Reis de Azevedo
Travessa Percy Withers 88 apto 51,Água Verde
80240190 Curitiba-PR

Of. DPH - 01/2005 Brasília, 06 de junho de 2005

Processo: 474790/2004-5 Com[illegible] de Ass[illegible]mento: MS Mod[illegible]

Referência: Edital CNPq nº 019/2004 - Universal
Instituição: Departamento de Odontologia / Pontifícia Universidade Católica do Paraná
Projeto: Efeitos de drogas psicotrópicas e da pilocarpina sobre glândulas parótidas de ratos. Análises histomorfométrica, sialométrica e imunohistoquímica

Com base na recomendação do Comitê de Assessoramento e de acordo com o estabelecido no **Edital CNPq nº 019/2004 - Universal**, a Diretoria Executiva do CNPq aprovou a concessão do auxílio financeiro no montante abaixo discriminado. A aplicação dos recursos financeiros deverá ser realizada no prazo máximo de 24 (vinte e quatro) meses a partir da primeira liberação.

Valor Aprovado: R$ 21.244,10

No anexo 1 estão as orientações para acesso e emissão do Termo de Concessão e Aceitação e para informação da conta vinculada. No anexo 2 está a solicitação de abertura de conta corrente tipo "B" do Tesouro Nacional.

Após a leitura das condições gerais e preenchimento dos itens em branco (exceto data), solicitamos encaminhar ao CNPq três vias, assinadas e rubricadas, do referido Termo dentro do prazo máximo de 15 (quinze) dias a contar desta correspondência. Uma via será restituída após a assinatura pelo CNPq. Caso V. Sa. não devolva o Termo no prazo estipulado, o auxílio será cancelado.

A prestação de contas e o relatório técnico do projeto devem ser apresentados ao CNPq até 60 (sessenta) dias após o término das atividades. Advertimos que a existência de pendência técnica (relatório técnico não remetido) ou financeira (prestação de contas vencida referente a auxílios anteriores), impedem a implementação de auxílios.

Colocamo-nos à disposição para prestar outros esclarecimentos, lembrando que as correspondências deverão ser remetidas para o endereço abaixo, com o nome completo de V.Sa. e número do processo a que se refere.

Atenciosamente,

MANOEL BARRAL NETTO
Diretor de Programas Temáticos e Setoriais

Endereço: SEPN QD.509 BLOCO A - ED.NAZIR I - SALA 105, BRASILIA, DF, 70750501
E-mail: COSAU@CNPQ.BR
ANEXO 1: Orientação para emissão do Termo de Concessão e Aceitação, e para informação da conta vinculada.
ANEXO 2: Solicitação de abertura de conta tipo "B".
OBS: O manual de Prestação de Contas encontra-se a disposição de V.Sa. no site do CNPq. http://www.cnpq.br/formularios/formgerais.htm.
