# Supplementary figures and images for: Effects of Benzodiazepines on Acinar and Myoepithelial Cells
Source: Front Pharmacol. 2016 Jun 24;7:173. doi: 10.3389/fphar.2016.00173 (PMC4919344; doi:10.3389/fphar.2016.00173)

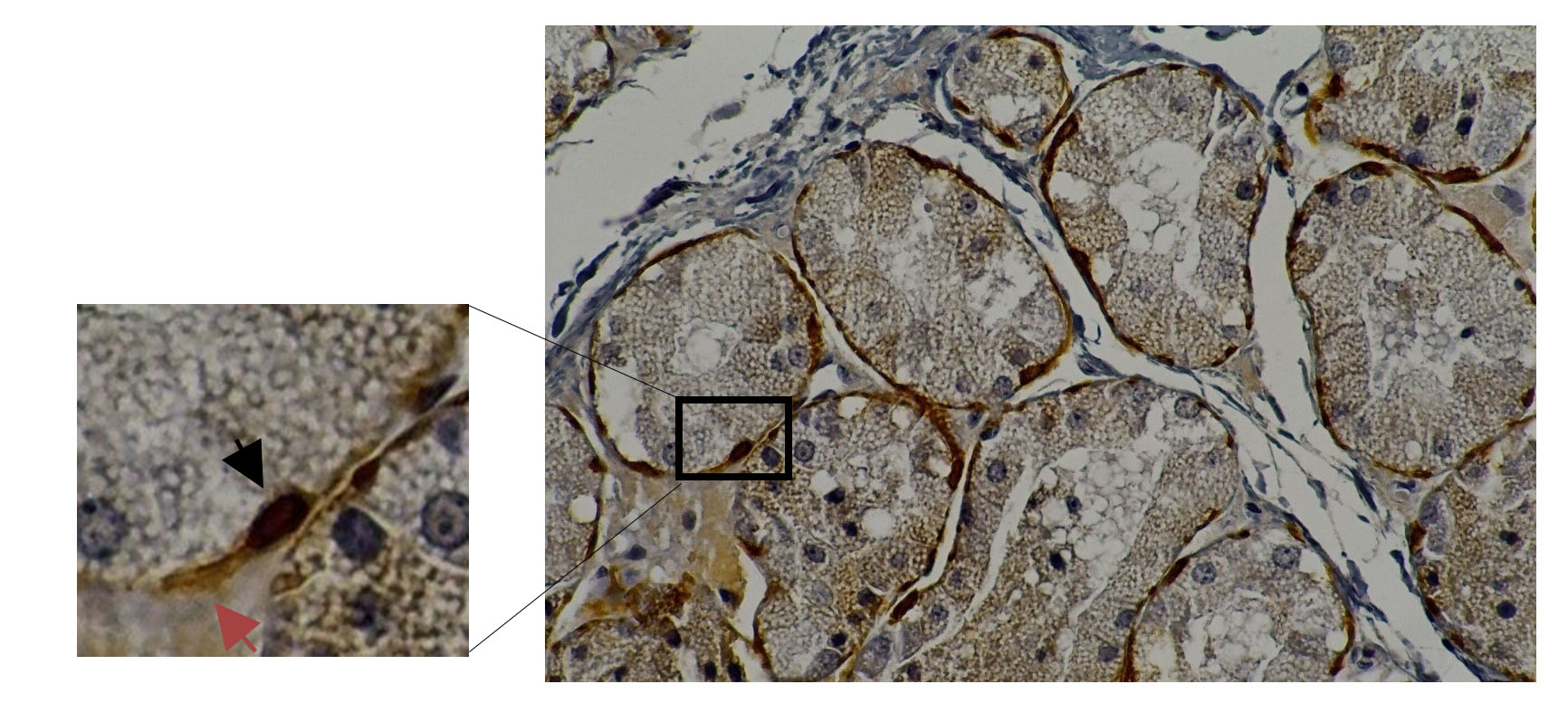

Supplement: Supplementary file 3 [file Image1.JPEG]
